# Children’s exposure to cocaine detected by hair analysis: a systematic review and meta-analysis

**DOI:** 10.1186/s12887-025-06146-x

**Published:** 2025-10-21

**Authors:** Clara Cestonaro, Massimo Carollo, Alessia Russo, Anna Aprile, Donata Favretto, Claudio Terranova

**Affiliations:** 1 Department of Women’s and Children’s Health, University of Padova, Padova, Italy; 2 Legal Medicine and Toxicology Unit, University Hospital of Padova, Padova, Italy; 3 Department of Diagnostics and Public Health, University of Verona, Verona, Italy; 4 Legal Medicine, Department of Diagnostics and Public Health, University of Verona, Verona, Italy; 5 Department of Cardiac, Thoracic, Vascular Sciences and Public Health, University of Padova, Padova, Italy

**Keywords:** Children, Cocaine, Hair analysis

## Abstract

**Background:**

Cocaine is a widespread drug of abuse to which children can also be exposed. The modes of exposure may vary depending on the age of the child. In addition to blood and urine analysis, hair analysis is currently used in some clinical contexts to investigate children’s exposure to drugs of abuse, including cocaine. This systematic review aims to collect data on children’s exposure to cocaine proven by hair analysis, and to understand whether the concentration of cocaine in the hair of children varies with age, potentially providing insights into the nature of their exposure.

**Methods:**

This review was conducted in PubMed (including PubMed Central and Medline), Web of Science (Core Collection), and grey literature databases including Web of Science Preprint Citation Index, OpenGrey.eu, and Grey Literature Report, from inception until July 15, 2024. Cross-sectional studies, case series, and case reports where cocaine was detected in children’s hair using mass spectrometry techniques, and concentrations reported, were included. Study selection and data extraction were conducted independently by two reviewers.

**Results:**

The systematic review included 21 studies. Ten studies were eligible for meta-analysis. Cocaine median concentrations (ng/mg) at hair analysis were 1.17 [95% CI (0.19, 7.17)] in children under one year and 0.39 [95% CI (0.13, 1.13)] in children over one year. The heterogeneity tests indicated high between-study heterogeneity (I2 = 93.76% in studies including children under one year; I2 = 93.92% in studies including older children).

**Conclusions:**

The finding of higher median concentration of cocaine in hair of infants under one year should be cautiously read, considering the characteristics of hair of young children and the influence of possible exposure occurring even or only in utero. The heterogeneity between the studies suggests differences in the study populations, and these may be reflected in terms of exposure levels. Given this heterogeneity and the potential for publication bias, further research involving larger populations and employing more rigorous methodologies is essential. The assessment of these cases cannot therefore be based on hair analysis alone, but must consider the analysis of other biological matrices, the history of the child and family, and information on the living environment.

**Supplementary Information:**

The online version contains supplementary material available at 10.1186/s12887-025-06146-x.

## Background

Cocaine is one of the most widespread drugs of abuse. According to the 2022 World Drug Report of the United Nations Office on Drugs and Crime (UNODC) [1], an estimated 21.5 million people used cocaine in 2020, with North America and Europe being the two main consumer markets. In 2022, the global number of users increased to 23.5 million [2]. Extracted from *Erythroxylon coca* dried leaves, cocaine acts as a psychostimulant by inhibiting the reuptake of noradrenaline, dopamine, and serotonin, and can be used through sniffing, smoking, or intravenous injection [3–5]. Children can also be exposed to this substance, and the modes of exposure may vary depending on age, including in-utero, breastfeeding, passive inhalation, accidental ingestion, and intentional administration [6]. Moderate doses of cocaine cause a euphoric effect, decrease appetite, and fatigue. The most severe neurological consequences of cocaine use include ischemic stroke, intracerebral hemorrhage, and convulsions [5]. In the cardiovascular system, cocaine is associated with acute toxicity, inducing arrhythmias, acute hypertension, coronary spasm, acute myocardial infarction, and was also described as associated with more chronic effects such as cardiomyopathy and coronary artery disease [7]. During pregnancy, cocaine interacts with the placenta, which expresses both serotonin and norepinephrine transporters, leading to uterine contraction, vasoconstriction, and resulting in premature delivery, decreased placental blood flow, and intrauterine growth retardation [4]. Fetal exposure to cocaine has been demonstrated to affect head circumference, length, and birth weight [8].

Blood and urine analysis allow the investigation of acute or recent exposures to cocaine use, whereas hair analysis enables the detection of more prolonged exposures [9]. Hair analysis received increasing attention over the past decades thanks to its non-invasive sampling and long detection window, and it is currently used not only in forensic science but also in clinical toxicology. The rationale of this method is that drugs circulating in the bloodstream are incorporated into hair cells, but positive findings can also result from diffusion from sweat or other secretions and from external contamination [9]. For some drugs of abuse, including cocaine, the Society of Hair Testing (SoHT) indicated cut-off values concerning the concentration of the substance in mg of hair that allow the identification of drug users. However, these cannot be considered in children, where a lower limit of quantification is required [10] and no age-specific reference concentrations exist.

In view of the prevalence of cocaine use among adults, resulting in a risk of children's exposure, and because hair analysis is increasingly used in both forensic and clinical contexts, it would be useful to deepen the subject of its interpretation in children. This would require the systematic comparison of data gathered from different contexts. Currently, however, most of the studies on children’s exposure to cocaine provide an epidemiological picture limited to a reference context, and recent systematic reviews are lacking. Therefore, an updated systematic review and meta-analysis was deemed necessary to identify and analyze all available data on children’s exposure to cocaine, and to understand whether the concentration of cocaine in the hair of children varies with age, potentially providing insights into the nature of their exposure.

## Methods

### Literature search strategy and selection criteria

This systematic review was conducted in accordance with the methodological guidance of the *Cochrane Handbook for Systematic Reviews of Interventions* (version 6.5, 2024) [11] and reported following the Preferred Reporting Items for Systematic Reviews and Meta-Analyses (PRISMA) statement. The study protocol was registered on PROSPERO (CRD42024510195).

The review question was framed using an adapted PEO approach:

- Population (P): children (aged 0–18 years) underwent hair analysis to investigate exposure to drugs of abuse;
- Exposure (E): cocaine exposure assessed by hair analysis performed using validated methods for the detection and quantification of cocaine;
- Outcome (O): reported concentration of cocaine in hair (ng/mg).

Studies evaluating and reporting cocaine hair concentrations in pediatric cohorts were searched in the bibliographic databases PubMed (including PubMed Central and Medline), Web of Science (Core Collection), and grey literature databases including Web of Science Preprint Citation Index, OpenGrey.eu, and Grey Literature Report, from inception until July 15, 2024. The search terms were related to exposure to cocaine and hair analysis in the pediatric population. The detailed search strategy is provided in Additional file 1. All types of studies were searched. Narrative or systematic reviews and meta-analyses, as well as book chapters, editorials, and conference abstracts, were excluded but screened to identify other potential studies to be included. Cross-sectional studies, case series, and case reports were included. After removing duplicates from the five different databases, one legal medical doctor (CC) and one clinical pharmacologist and toxicologist resident doctor (MC) screened individually the titles and abstracts of records identified to remove articles that were clearly irrelevant. The full texts of the selected articles were then reviewed by the two authors (CC, MC) to define whether they met the inclusion criteria, consisting of cocaine detection and quantification in children's hair performed using mass spectrometry techniques. Any disagreements among evaluators were resolved through discussion or, if consensus was not yet reached, through the intervention of a third evaluator (DF/CT).

### Data extraction

Data from each article included in the systematic review was independently extracted by two reviewers (CC and MC). Any discrepancy was resolved through discussion or a third (CT) intervention. For each article, data on the following items was retrieved:

- characteristics of the study: author(s), year of publication, country in which the study was conducted or, where not available, author affiliation, number of children enrolled, age of children;
- outcomes: number of children positive for cocaine at hair analysis, concentration in ng/mg of cocaine and benzoylecgonine at hair analysis.

Studies or subgroups of patients were categorized into two age-based groups considering the possible nature of exposure to cocaine: *Group A* (children within one year of age, whose exposure could have been *only* or *also* in-utero) and *Group B* (children older than one year, whose positivity at hair analysis cannot be related to an in-utero exposure).

### Statistical analysis

We performed one-sample meta-analyses of medians using the quantile estimation (QE) method under a random effects model [12]. Only studies with a low or moderate risk of bias were included in the meta-analysis to improve comparability. The QE method facilitates the aggregation of study-specific median estimates, adjusting for sample sizes and heterogeneity across studies. Our analysis aimed at estimating each age-based subgroup's pooled median effect size, considering the between-study variability inherent in the random effects model. Prior to meta-analysis, values equal to zero were replaced with 0.0001 to allow for natural logarithmic transformation and prevent undefined computations. All outcome measures were then transformed to the log scale to address right-skewness and to ensure consistency in scale across studies. After meta-analysis, pooled estimates and their 95% confidence intervals (CIs) were back-transformed to the natural scale (ng/mg) for interpretability. Study weights reflect variance estimates in the log domain.

The between-study variance (τ2) was calculated using the Hunter-Schmidt estimator with a small sample size correction for *Group A* and the Paule–Mandel (PM) estimator for *Group B*, to provide a robust measure of heterogeneity [13–15]. Furthermore, the τ^2^, I^2^, and the Q test statistics were used to assess between-study heterogeneity. The τ^2^ represents the estimated variance of underlying effect sizes across studies, whereas Q test *p*-value < 0.05 and I^2^ > 40% indicate the presence of heterogeneity. To evaluate the presence of publication bias, funnel plots showing the individual observed study outcome (on the x-axis) against the corresponding standard error (on the y-axis) were reported for both *Group A* and *Group B* meta-analyses and analyzed by using Egger's test.

All statistical analyses were performed using the R software version 4.2.2 (packages “metamedian”, “metafor”, and “metadata”) [16].

### Risk of bias assessment

The quality of the included studies was independently assessed by two authors (CC and MC), using a modified Newcastle–Ottawa Scale for cross-sectional studies and, as for case reports, risk of bias was assessed using criteria as recommended by Nambiema et al., 2021 [17].

The modified Newcastle–Ottawa Scale for cross-sectional studies used in this systematic review is shown in Additional file 2. As for case reports, according to Nambiema et al., when assessing the risk of bias the following aspects should be considered: the context of the case report, the accuracy of assessment methods, the evaluation of potential confounders, and the presence of selective reporting.

## Results

### Study selection

**Fig. 1:**
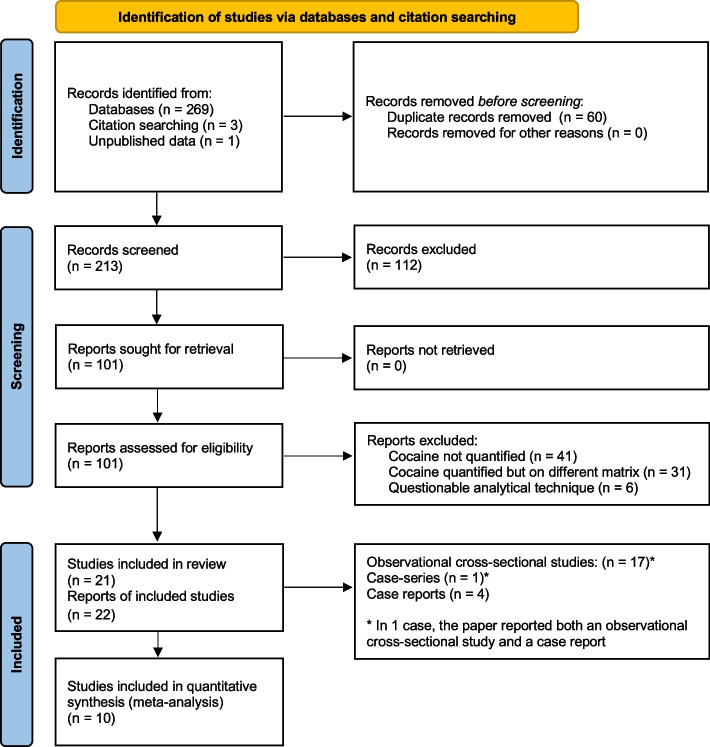
Flowchart of the study selection process

The PRISMA flowchart [18] summarizing the study selection process is shown in Fig. 1. The search strategy allowed the identification of a total of 269 articles, with an additional 3 studies being identified through snowball searching. One study of the present authors containing unpublished data, and still under submission, was included. After removing duplicates (*n* = 60), 213 articles remained for consideration. The title and abstract screening identified 101 studies for full-text reading. Of the articles whose full text was read, 22 met the eligibility criteria and were included in the systematic review, for a total of 21 studies (one of the 22 eligible studies [19] contained the same results concerning cocaine as another eligible study published by the same author, and thus was not included). Reasons for article exclusion consisted of lack of cocaine quantification (*n* = 41), quantification of cocaine but in a different matrix from children’s hair (*n* = 31), and use of methods different from mass spectrometry techniques (*n* = 6). The included studies are summarized in Additional file 3.

### Study characteristics

The articles were published between 1994 and 2024. Of the 21 included studies, 16 consisted of observational studies, 4 of case reports, and 1 consisted of both an observational study and a case-series [20].

Children included in the studies ranged from neonates to 18 years old. A total of 530 children positive for cocaine at hair analysis was found; 93 of 530 were younger than one year, and 437 were older.

Concentrations of cocaine and benzoylecgonine in hair ranged from the lower limit of quantification (LOQ) to 290 ng/mg and 55.4 ng/mg, respectively.

### Descriptive results

#### Neonates and infants within their first year

The earliest study included in the review was conducted by Di Gregorio et al. (1994) [21] on pregnant women without prior prenatal care and their neonates. Among these neonates, 22 tested positive for cocaine via hair analysis, with concentrations ranging from 0.8 to 243.5 ng/mg (median: 16.80 ng/mg).

In the early 2000s, while investigating toxicological factors predicting the occurrence and severity of withdrawal syndromes in neonates born to drug-using mothers, Vinner et al. [22] identified two neonates positive for cocaine via hair analysis. One neonate had levels below the limit of quantification (LOQ, < 2 ng/mg), while the other had a concentration of 17.9 ng/mg.

Two years later, Garcia-Algar et al. [23] described an 11-month-old breastfed child admitted to the Emergency Department (ED) with apparent generalized seizures. Urine analysis was positive for methylenedioxymethamphetamine (MDMA), while hair analysis revealed cocaine concentrations of 1.3 ng/mg in the proximal segment and 2.95 ng/mg in the distal segment.

In 2011, Joya et al. [24] described a 1-month-old breastfed boy who presented to the ED with respiratory failure, cyanosis, myosis, and hypotonia. Hair analysis detected opiates and cocaine at a concentration of 17.5 ng/mg.

More recently, Franz et al. [20] reported six children under one year of age whose hair tested positive for cocaine, with concentrations ranging from 0.01 to 5.40 ng/mg (median: 0.07 ng/mg). Bertaso et al. [25], in their investigation of suspected in-utero exposure to drugs of abuse, found cocaine in the hair of 19 neonates, with concentrations up to 38.6 ng/mg. Similarly, Cestonaro et al. [26] reported on 42 infants under one year of age whose hair tested positive for cocaine, with a maximum concentration of 290 ng/mg and a median concentration of 0.655 ng/mg.

#### Children over one year and adolescents

In 2010, Papaseit et al. [27] described the case of a 13-month-old child of parents undergoing methadone treatment who exhibited signs of acute intoxication from methadone. Hair analysis also revealed the presence of cocaine at a concentration of 17.24 ng/mg.

Previously, De Giorgio et al. [28] reported the case of a 6-year-old child admitted to the Emergency Department (ED) with generalized malaise, agitation, tachycardia, arterial hypertension, and mydriasis. Urine samples tested positive for cocaine, and hair analysis showed mean cocaine levels of 16 ng/mg. The authors hypothesized that involuntary environmental exposure resulted in passive but significant intake of the substance.

Passive exposure to cocaine was also described by Smith and Kidwell [29], who conducted a study on adults in rehabilitation programs. These adults had lived with at least one child aged 1–14 years during the previous 90 days. Hair analysis revealed that 23 of the children tested positive for cocaine, with concentrations reaching up to 14.4 ng/mg.

Franz et al. [20] reported on five siblings aged 1–8 years whose hair analysis showed cocaine concentrations ranging from 0.088 to 3.25 ng/mg. Notably, the concentrations were higher in two of the younger children, likely due to their closer contact with their drug-using mother. A subsequent hair analysis six months later found cocaine detectable in only one sibling, likely reflecting the removal of the children from their mother’s care.

In the same study [20], Franz et al. analyzed a larger cohort of children and adolescents up to 16 years old. Among those aged 1–6 years, 31 tested positive for cocaine at hair analysis, with a median concentration of 0.15 ng/mg (range: 0.02–20.0). Among children aged 6–14 years, 21 tested positive, with a median concentration of 0.10 ng/mg (range: 0.01–2.80). For adolescents aged 14–16 years, 17 tested positive, with a median concentration of 0.14 ng/mg (range: 0.03–15.0). The authors observed that the risk of exposure to drug-using caregivers decreases with age. However, higher concentrations in adolescent hair suggest potential accidental or deliberate use, possibly in addition to passive exposure.

In a study by the present authors that was still unpublished at the time of the literature search [30], cocaine concentrations in the hair of children up to 16 years were described. Median concentrations (in ng/mg) were 0.09 for toddlers aged 1– < 3 years, 0.116 for children aged 3– < 6 years, and 0.135 for children aged 6– < 12 years. The results of this study suggest that exposure to drugs of abuse is a non-negligible problem, particularly in infants and toddlers.

The adolescents’ deliberate intake of cocaine was contemplated by Haas et al. [31], who analyzed the hair of some patients up to 16 years of age presenting to the ED with alcohol intoxication: two tested positive for cocaine in blood and hair, the latter with concentrations of 0.044 and 1.5 ng/mg.

By studying children hospitalized for a presumed intoxication, Alvarez et al. [32] described a 34-months-old child accidentally poisoned with buprenorphine who tested also positive for cocaine at hair analysis, with concentration of 0.013 ng/mg in the proximal hair segment, and a 11-years child living in a context of domestic violence (consisting of drug-facilitated sexual assaults of the mother perpetrated by the father) with 0.2 ng/mg of cocaine in proximal hair segment.

Joya et al. [33] analyzed the hair of preschoolers (age 18 months-5 years) presenting to the emergency room for a variety of general medical complaints, without signs or symptoms suggestive of exposure, and found 21 positives for cocaine with concentrations of 0.3–5.96 ng/mg. According to the authors, the results showed evidence that preschoolers can be exposed to drugs from environmental sources, being the route probably linked to parental active or passive exposure.

The hair of children admitted to the ED was also analyzed by Pichini et al. [34, 35], who described two different cohorts: respectively, the hair of 43 and 23 children tested positive for cocaine with median concentrations of 0.32 ng/mg and 0.54 ng/mg. The results of the investigations, which included several drugs of abuse, were considered to confirm a high prevalence of unsuspected exposures in the reference setting.

Almost ten years later than Pichini, Garcìa-Caballero et al. [36] reported on hair analysis of children attending emergency services from 2009 to 2021. These authors found 12 children aged 1–6 years positive for cocaine with concentrations ranging from < 0.25 ng/mg to 28.67 ng/mg (median 11.20 ng/mg). In general, most positive cases of this study corresponded to children younger than 3 years old, suggesting a greater vulnerability of young children to drug consumption environments.

In 2013, Pragst et al. [37] investigated hair samples from children aged 1–16 years living with parents using methadone or suspected of abusing illegal drugs and observed that cocaine was the most frequently detected drug, being positive in 73 children, with concentrations up to 17.8 ng/mg. More recently, Pragst et al. [38] analyzed hair samples from family members involved in a social support project dealing with drug use: the minimum age of children was one year to avoid prenatal drug incorporation in hair. The authors found 18 children aged 7–14 years positive for cocaine, with concentrations ranging from 0.02 to 1.24 ng/mg.

Himes et al. [39], comparing six-year-old-children prenatally exposed to methamphetamine with non-exposed children, despite primary caregivers reporting no cocaine use, found respectively 6 and 3 children positive for cocaine at hair analysis with concentrations of 0.039–0.315 ng/mg (median 0.062 ng/mg) and of 0.074–0.195 ng/mg (median 0.102 ng/mg). Stauffer et al. [40], by reviewing hair analysis of children up to 17.5 years who underwent testing in the context of suspected child abuse evaluation, found a total of 37 positives for cocaine with a maximum concentration over 10 ng/mg. It is, however, important to note that in these two latter studies [39, 40], the ChildGuard test was used, which does not include hair washing to improve detection of environmental exposure.

### Group A and Group B one-sample meta-analysis of hair cocaine concentrations

It was possible to include studies in the meta-analysis if the sample size was equal to or greater than 3 individuals. Furthermore, data on the median, first quartile (Q1) and third quartile (Q3), and/or minimum and maximum values were required. Studies considered as having an overall high risk of bias were not included.

**Fig. 2:**
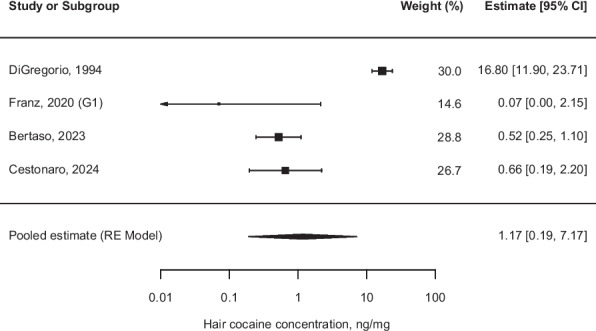
Forest plot of cocaine hair concentrations (ng/mg) one-sample meta-analysis of *Group A* (infants of 0–1 year). ${\tau }^{2} = 2.8240 \;(\text{SE}=1.6471)$; $\uptau = 1.6805$; ${I}^{2} = 93.76\text{\%}$; $\text{Q }(\text{df }= 3) = 92.2131, p< 0.001$; Abbreviations: *CI* Confidence interval; *RE* Random effect

**Fig. 3:**
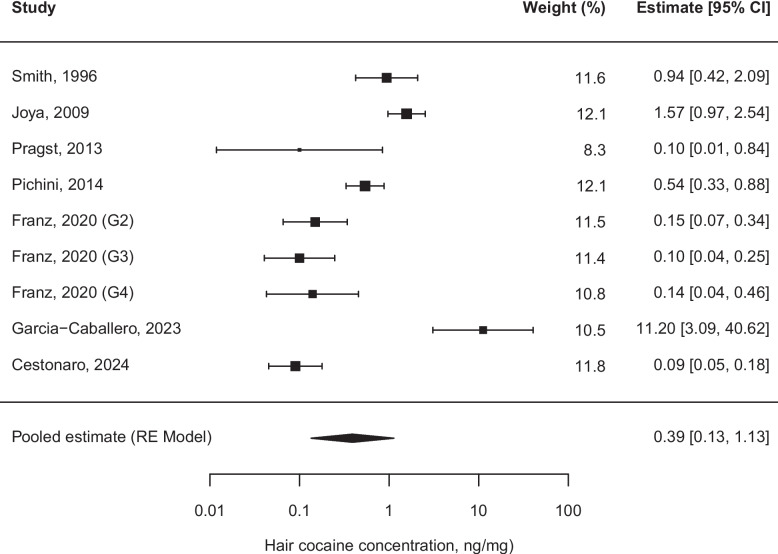
Forest plot of cocaine hair concentrations (ng/mg) one-sample meta-analysis of *Group B* (children over 1 year old and adolescents up to 18 years)*.* ${\tau }^{2} = 2.3744 \;(\text{SE }= 1.3250)$; $\uptau = 1.5409$; ${I}^{2}=93.92$; $\text{Q }(\text{df }= 8) = 97.5014, p< 0.001$; Abbreviations: *CI* Confidence interval; *RE* Random effect

In *Group A*, four studies were included, with an estimated median of 1.17 [95% CI (0.19, 7.17)] (Fig. 2). In *Group B*, seven studies were included, with an estimated median of 0.39 [95% CI (0.13, 1.13)] (Fig. 3). The heterogeneity tests indicated high between-study heterogeneity, with I^2^ values of 93.76% and 93.92%, respectively.

### Publication bias assessment

According to Egger's test, publication bias was observed for *Group A* meta-analysis (*p* = 0.043, Z = −2.03). As for *Group B* meta-analysis, publication bias was not found (*p* = 0.73, Z = −0.34). Funnel plots are shown in Additional file 4 and Additional file 5.

### Risk of bias assessment

Among the 17 cross-sectional studies included in this systematic review, 2 (11.7%) were considered to have a high risk of bias (Additional file 6 and Additional file 7) corresponding to one between: selection—comparability—reporting/confounding.

Regarding the case reports, three were assessed as having a low risk and two as moderate risk of bias (Additional file 8 and Additional file 9).

## Discussion

To the best of our knowledge, this is the first comprehensive systematic review focusing on cocaine concentration in hair analysis of children and investigating whether it varies with age, providing insights into the nature of the exposure. Previously, in 2015 Wang et al. [6] published a systematic review of the literature that reported on the hair concentrations of various drugs in children, including cocaine. Fifty-two articles were selected, and results were further aggregated in categories according to the type of exposure (i.e., babies under 1 month with in-utero or breast milk exposure, children with proven active exposure, children with possible active exposure, and children with likely passive exposure). Almost all children positive for cocaine and benzoylecgonine were identified as likely to be passively (e.g., from smoke or sweat/sebum) or in-utero exposed. The authors found higher concentrations in cases of in-utero exposure compared to passive exposure, but a considerable overlap in concentrations was observed, and as only one child was likely actively exposed, they considered it unclear whether cocaine and/or benzoylecgonine concentrations in hair could discriminate active exposure from environmental exposure [6].

The ways in which a child can be exposed to an illicit drug are manifold and influenced by the age of the child and the characteristics of the substance. During pregnancy, the transfer of drugs of abuse across the placenta depends on the physical properties of the compound, such as molecular size, lipophilicity, pKa, and blood pH [41]. With regard to cocaine, the transport mechanisms may vary at different pregnancy stages, and previous literature described that the placenta metabolizes this substance and that myometrium and placental membranae store it [42]. After birth, children can be exposed to drugs of abuse through lactation: breastfeeding provides nutrients to the child, and at the same time also transfers environmental chemicals and substances of abuse, including cocaine. Previous literature describes substantial variability in the levels of cocaine concentrations detected in breast milk, which might reflect methodological issues in the detection of the substance, interindividual variability in pharmacokinetics, and differences in the amounts of drug used [43]. In view of its half-life, however, it is assumed that cocaine passes rapidly into breast milk, where, according to animal and human studies, it can be detected at high concentrations [44].

Children can also passively inhale smoked drugs in quantities that may be increased in younger ones who have higher respiratory rates. Passive exposure to crack-cocaine smoke, a form of the substance quickly absorbed by the pulmonary circulation, and which rapidly reaches the central nervous system, was deemed to occur in a similar way to second-hand cigarette smoke [45].

Accidental exposure represents another possible non-negligible issue, in particular in toddlers able to explore their environment, and who show hand-to-mouth behaviors [46, 47].

The high incidence of hospitalizations among children aged 1 to 3 years due to intoxications is often attributed to their natural tendency to explore their environment. Dinis-Oliveira and Magalhães [48] described how unintentional, non-neglectful intoxications—those resulting from accidents despite adequate precautions and supervision—should be distinguished from neglectful cases, where a child’s needs are not met, and caregivers engage in risky behaviors, whether consciously or not.

The same authors also highlighted that child intoxication can be intentional or abusive and can be linked to caregiver psychopathology. In some cases, substances may be used as a ‘disciplinary’ method to modify behavior or even facilitate sexual abuse. Poisoning in children represents an especially insidious form of maltreatment, necessitating vigilant oversight and robust preventive measures.

Finally, as individuals approach adolescence, exposure to illicit substances increasingly reflects patterns of deliberate consumption, underscoring the need for targeted intervention and education during this critical developmental stage.

In 2019, the European School Survey Project on Alcohol and Other Drugs found a lifetime prevalence of cocaine use at age of 16 up to 6.3% for boys and 2.8% for girls (mean 2.2% and 1.6%, respectively), with an average of 0.4% students reporting the use of cocaine/crack at age of 13 or younger [49].

When trying to trace the probable modes of exposure to cocaine in children who tested positive for this substance at hair analysis, age is therefore a non-negligible factor. Depending on age, the likelihood of a route of exposure will differ, consider for instance breastfeeding (children should be exclusively breastfed for the first six months of life and breastfeeding should be continued for up to two years of age or beyond [50]), accidental ingestion (toddlers desire to explore new objects), and deliberate use (adolescents may try alcohol and drugs).

In the present analysis, children were categorized into two age-based groups considering the possible nature of exposure to cocaine: infants within one year of age, whose exposure could have been *only* or *also* in-utero, and children older than one year, whose positivity at hair analysis cannot be related to an in-utero exposure. Indeed, as reported by Pragst et al. [38], prenatal hair is completely replaced between 6 and 12 months after birth; therefore, in utero exposure can be detected in hair up to one year of age, while a positive result in children older than one year reflects exposures occurring after birth.

Due to the small number of studies included in the meta-analysis, no further age groups could be studied. The meta-analysis revealed a higher pooled median concentration of cocaine in hair among infants under one year [1.17 ng/mg; 95% CI (0.19, 7.17)] compared to older children and adolescents [0.39 ng/mg; 95% CI (0.13, 1.13)]. In *Group A*, the potential contribution of in utero exposure must be considered, especially within the first months of life, while *in Group B*, hair positivity reflects postnatal exposures, including passive inhalation, accidental ingestion, or, in some cases in adolescents may also reflect voluntary use.

The trend to higher concentrations in infants under one year (*Group A*) herein observed should be cautiously read, taking also into account the characteristics of young children’s hair, which is finer and more porous, thus more prone to contamination [51]. Moreover, the CI is very large, likely due to the limited number of studies included (only four) and the results from the study by Di Gregorio et al [21]. The narrower confidence interval observed in *Group B* may indicate more consistent postnatal exposure patterns or methodological uniformity across studies.

Differences in the study populations (e.g., at-risk populations, specific environments) may be reflected in terms of exposure levels, thus affecting the results. Unfortunately, most of the included articles did not systematically report variables such as socioeconomic status and mothers' or caregivers' drug use patterns, preventing their inclusion in the analysis. Also, a meta-regression to explore sources of heterogeneity was not possible due to the limited number of studies included in the meta-analysis. As such, it is not possible to draw universal conclusions about the relation between cocaine concentration at hair analysis and age, nor to draw conclusions about the nature of exposure.

Although this systematic review suggests that in infants (age < 1 year) higher concentrations of cocaine in hair may be found, that can be influenced by in utero exposure, pediatricians should be aware that this should not preclude the collection of in-depth information, the request for further analysis (including repeat hair analysis at a later date) and discussion with a forensic toxicologist. Considering the possible implications of reporting these cases to authorities, including in terms of child custody, it is essential to trace a comprehensive framework that allows for the differentiation of cases where exposure was unintentional and non-neglectful (e.g. breastfeeding) and cases where a voluntary administration occurred or where the accidental contact was attributable to neglect (for example, because caregivers left the substance at home, accessible to the child). Clinicians who take care of children with exposure to cocaine, proven by hair analysis, should remember that in order to trace the mode of exposure it is essential to consider not only the result of the hair analysis, but also the age of the child and its psychomotor development, anamnestic data, information on family and living environment, results of other toxicological analyses which allow the identification of recent systemic exposures (blood, urine). Hair analysis of caregivers and siblings can also be useful in this view.

### Strength and limitations

A strength of this systematic review is that it is the first systematic review of the literature focusing on cocaine concentration in hair considering the age of children, which relates to the possible modes of exposure. Furthermore, we employed an innovative meta-analytical technique that allows for the meta-analysis of medians in one-sample studies. This approach has proven superior to the transformation-based methods (e.g., estimating sample means and standard deviations) for subsequent meta-analysis [52]. However, there are also some limitations. The scarcity of data, the heterogeneity across studies, and the possibility of publication bias necessitate cautious interpretation of the results. In particular, the publication bias observed in *Group A* should be interpreted cautiously as, in general, tests do not perform well when the number of included studies is less than ten [11]. These challenges underscore the imperative for more stringent research methodologies in this domain.

In this regard, prospective, multicenter cohort studies including well-defined pediatric populations exposed to cocaine under different circumstances (e.g., in utero, through breastfeeding, the environment, accidental ingestion or intentional administration) are advocated, as well as comparative studies integrating the results of hair, blood and urine analyses, together with detailed clinical, environmental and anamnestic data, and longitudinal studies to investigate changing patterns of exposure and potential health outcomes in exposed children.

Moreover, as substantial heterogeneity was observed in both age groups, a meta-regression could have been a valuable tool to further explore potential sources of heterogeneity. However, given the limited number of studies included in each subgroup (n = 4 for Group A, n = 7 for Group B), a meta-regression was not performed, in line with current methodological recommendations, which advise caution when fewer than 10 studies are available [11], due to the risk of overfitting and unreliable estimates. Future reviews with a broader evidence base may benefit from meta-regression analyses to better identify study-level effect modifiers.

## Conclusions

In view of the prevalence of cocaine use among adults, children’s exposure represents an emergent issue. Considering the multiple modes of exposure, it would be useful to deepen the subject of the interpretation of children's hair analysis, and this would require the comparison and critical evaluation of a large amount of information from different contexts.

Through the identification and analysis of data on children’s exposure to cocaine proven by hair analysis, this systematic review and meta-analysis revealed a higher median concentration of cocaine in hair of infants under one year of age, where the potential contribution of in utero exposure must be considered, compared to older children and adolescents, where positivity results from environmental passive exposure, accidental contact, or also intentional use. This finding should however be cautiously read, taking also into account the characteristics of hair of young children and the heterogeneity between the studies. The findings emphasize the need to integrate biological and contextual information to better understand the nature and implications of pediatric exposure to cocaine. At present, a comprehensive approach that integrates the results of hair analysis and of other matrices, along with history and clinical data from children and caregivers, is crucial for identifying the more probable modes of exposure in the actual cases with the aim of developing effective strategies to protect the health and the development of the child.

## Supplementary Information

Additional File 1. Detailed search strategy.

Additional File 2. Risk of bias assessment - modified Newcastle-Ottawa Scale for cross-sectional studies.

Additional File 3. Summary of included studies.

Additional File 4. Publication bias assessment – Funnel Plot, *Group A*.

Additional File 5. Publication bias assessment – Funnel Plot, *Group B*.

Additional File 6. Risk of bias assessment – Traffic Light Plot (.png).

Additional File 7. Risk of bias assessment – Summary Plot (.png).

Additional File 8. Risk of bias assessment – Traffic Light Plot (.png).

Additional File 9. Risk of bias assessment – Summary Plot (.png).

## Data Availability

Data analyzed during this systematic review are included in the manuscript and its additional files.
