# Supplementary material for: Children’s exposure to cocaine detected by hair analysis: a systematic review and meta-analysis
Source: BMC Pediatr. 2025 Oct 21;25:839. doi: 10.1186/s12887-025-06146-x (PMC12542512; doi:10.1186/s12887-025-06146-x)
Supplement: Supplementary file 1 — Additional File 1. Detailed search strategy. [file 12887_2025_6146_MOESM1_ESM.docx]

**Additional file 1.** Search strategy overview.

| **Search number** | **Query** | **Results** |
| --- | --- | --- |
| **PubMed (including PubMed Central and Medline)** | | |
| #1 | "cocaine"[MeSH Terms] OR "cocaine"[All Fields] OR "cocain*"[All Fields] OR "cocaine s"[All Fields] OR "cocaines"[All Fields] OR "cocainics"[All Fields] OR "cocaine related disorders"[MeSH Terms] OR "crack cocaine"[MeSH Terms] | 48.253 |
| #2 | "infant"[MeSH Terms] OR "Infant, Newborn"[MeSH Terms] OR "child"[MeSH Terms] OR "child, preschool"[MeSH Terms] OR "child*"[Title/Abstract] OR "juvenile*"[Title/Abstract] OR "newborn*"[Title/Abstract] OR "neonate*"[Title/Abstract] OR "toddler*"[Title/Abstract] OR "infant*"[Title/Abstract] OR "adolescent"[MeSH Terms] OR "adolescent*"[Title/Abstract] OR "youth*"[Title/Abstract] OR "pediatr*"[Title/Abstract] OR "paedriatr*"[Title/Abstract] OR "pediatrics"[MeSH Terms] OR "baby"[Title/Abstract] OR "babies"[Title/Abstract] | 4.863.832 |
| #3 | "hair analysis"[MeSH Terms] OR "hair analysis"[All fields] OR "hair sample testing"[All fields] OR "hair testing"[All fields] | 3.314 |
| #4 | "forensic toxicology"[MeSH Terms] OR "toxicolog*"[Title/Abstract] | 107.086 |
| #5 | "hair"[Title/Abstract] OR "hair"[MeSH Terms] | 100.987 |
| #6 | #4 AND #5 | 1.337 |
| #7 | #3 OR #6 | 4.214 |
| #8 | #1 AND #2 AND #7 | 106 |
| **Web of Science Core Collection** | | |
| #1 | ALL=(cocaine) OR ALL=(cocaines) OR ALL=(cocainics) OR ALL=(cocain*) OR ALL=("cocaine s") OR KP=("cocaine related disorders") OR KP=("crack cocaine") | 64.634 |
| #2 | TS=(infant*) OR TS=(child*) OR TS=(children) OR TI=(juvenile*) OR AB=(juvenile*) OR TS=(newborn*) OR TI=(neonate*) OR AB=(neonate*) OR TS=(adolescent*) OR TI=(youth*) OR AB=(youth*) OR TI=(toddler*) OR AB=(toddler*) OR TI=(baby) OR AB=(baby) OR TI=(babies) OR AB=(babies) OR TI=(pediatr*) OR AB=(pediatr*) OR TI=(paediatr*) OR AB=(paediatr*) OR KP=(pediatrics) OR KP=(paediatrics) | 3.444.065 |
| #3 | KP=("hair analysis") OR ALL=("hair analysis") OR ALL=("hair sample testing") OR ALL=("hair testing") | 2.471 |
| #4 | KP=("forensic toxicology") OR TI=(toxicolog*) OR AB=(toxicolog*) | 81.192 |
| #5 | TI=(hair) OR AB=(hair) OR KP=(hair) | 121.836 |
| #6 | #4 AND #5 | 1.174 |
| #7 | #3 OR #6 | 3.261 |
| #8 | #1 AND #2 AND #7 | 163 |
| **Web of Science Preprint Citation Index** | | |
| #1 | TS=(cocaine) OR TS=(cocaines) OR TS=(cocainics) OR TS=(cocain*) OR WC=(cocain*) OR TS=("cocaine s") OR AK=("cocaine related disorders") OR AK=("crack cocaine") | 333 |
| #2 | TS=(infant*) OR TS=(child*) OR TS=(children) OR TI=(juvenile*) OR AB=(juvenile*) OR TS=(newborn*) OR TI=(neonate*) OR AB=(neonate*) OR TS=(adolescent*) OR TI=(youth*) OR AB=(youth*) OR TI=(toddler*) OR AB=(toddler*) OR TI=(baby) OR AB=(baby) OR TI=(babies) OR AB=(babies) OR TI=(pediatr*) OR AB=(pediatr*) OR TI=(paediatr*) OR AB=(paediatr*) OR AK=(pediatrics) OR AK=(paediatrics) | 17.003 |
| #3 | AK=("hair analysis") OR TS=("hair analysis") OR WC=("hair analysis") OR TS=("hair sample testing") OR WC=("hair sample testing") OR TS=("hair testing") OR WC=("hair testing") | 3 |
| #4 | AK=("forensic toxicology") OR TI=(toxicolog*) OR AB=(toxicolog*) | 402 |
| #5 | TI=(hair) OR AB=(hair) OR AK=(hair) | 2.634 |
| #6 | #4 AND #5 | 5 |
| #7 | #3 OR #6 | 7 |
| #8 | #1 AND #2 AND #7 | 0 |
| **OPENGREY.EU - Grey Literature Database** | | |
| #1 | Any field (cocain* OR cocaine OR cocaines OR cocainics OR "cocaine s" OR "cocaine related disorders" OR "crack cocaine") | 33 |
| #2 | Any field (infant* OR child* OR children OR juvenile* OR newborn* OR neonate* OR adolescent* OR youth* OR toddler* OR baby OR babies OR pediatr* OR paediatr* OR pediatrics OR paediatrics) | 10.157 |
| #3 | Any field ("hair analysis" OR "hair sample testing" OR "hair testing") | 3 |
| #4 | Any field ("forensic toxicology" OR toxicolog*) | 76 |
| #5 | Any field (hair) | 481 |
| #6 | #4 AND #5 | 0 |
| #7 | #3 OR #6 | 3 |
| #8 | #1 AND #2 AND #7 | 0 |
| **Grey Literature Report** | | |
| #1 | Subject (cocaine) OR Keyword (cocaine) OR Title (cocaine) OR Subject (cocaines) OR Keyword (cocaines) OR Title (cocaines) OR Subject (cocainics) OR Keyword (cocainics) OR Title (cocainics) OR Subject (cocain*) OR Keyword (cocain*) OR Title (cocain*) OR Subject ("cocaine s") OR Keyword ("cocaine s") OR Title ("cocaine s") OR Keyword ("cocaine related disorders") OR Keyword ("crack cocaine") | 174 |
| #2 | Subject (infant*) OR Keyword (infant*) OR Title (infant*) OR Subject (child*) OR Keyword (child*) OR Title (child*) OR Subject (children) OR Keyword (children) OR Title (children) OR Subject (juvenile*) OR Keyword (juvenile*) OR Title (juvenile*) OR Subject (newborn*) OR Keyword (newborn*) OR Title (newborn*) OR Subject (neonate*) OR Keyword (neonate*) OR Title (neonate*) OR Subject (adolescent*) OR Keyword (adolescent*) OR Title (adolescent*) OR Subject (youth*) OR Keyword (youth*) OR Title (youth*) OR Subject (toddler*) OR Keyword (toddler*) OR Title (toddler*) OR Subject (baby) OR Keyword (baby) OR Title (baby) OR Subject (babies) OR Keyword (babies) OR Title (babies) OR Subject (pediatr*) OR Keyword (pediatr*) OR Title (pediatr*) OR Subject (paediatr*) OR Keyword (paediatr*) OR Title (paediatr*) OR Subject (pediatrics) OR Keyword (pediatrics) OR Title (pediatrics) OR Subject (paediatrics) OR Keyword (paediatrics) OR Title (paediatrics) | 16.291 |
| #3 | Subject ("hair analysis") OR Keyword ("hair analysis") OR Title ("hair analysis") OR Subject ("hair sample testing") OR Keyword ("hair sample testing") OR Title ("hair sample testing") OR Subject ("hair testing") OR Keyword ("hair testing") OR Title ("hair testing") | 9 |
| #4 | Subject ("forensic toxicology") OR Keyword ("forensic toxicology") OR Title ("forensic toxicology") OR Subject (toxicolog*) OR Keyword (toxicolog*) OR Title (toxicolog*) | 1.531 |
| #5 | Subject (hair) OR Keyword (hair) OR Title (hair) | 146 |
| #6 | #4 AND #5 | 1 |
| #7 | #3 OR #6 | 10 |
| #8 | #1 AND #2 AND #7 | 0 |
