# Supplementary material for: Children’s exposure to cocaine detected by hair analysis: a systematic review and meta-analysis
Source: BMC Pediatr. 2025 Oct 21;25:839. doi: 10.1186/s12887-025-06146-x (PMC12542512; doi:10.1186/s12887-025-06146-x)
Supplement: Supplementary file 2 — Additional File 2. Risk of bias assessment - modified Newcastle-Ottawa Scale for cross-sectional studies. [file 12887_2025_6146_MOESM2_ESM.docx]

**eTable 2**. Newcastle-Ottawa Scale adapted for cross-sectional studies.

| Criteria | Explanation | Scoring (0-2)* | |
| --- | --- | --- | --- |
| **Selection Bias** | Evaluates the approach to including participants exposed to cocaine. Recognizes that random selection is not feasible but looks for efforts to minimize bias in selecting participants (e.g., inclusion based on objective criteria). | 0 | High risk: no selection criteria |
|  |  | 1 | Moderate risk: selection criteria likely to introduce bias |
|  |  | 2 | Low risk: clear, objective criteria |
| **Comparability - Measurement Bias** | Assesses the comparability of results considering substance detection methods and procedures, including hair sampling collection. | 0 | High risk: procedure likely to introduce significant bias |
|  |  | 1 | Moderate risk: poorly or partially detailed method, or procedure likely to introduce some bias |
|  |  | 2 | Low risk: fully detailed analytical technique |
| **Reporting and Confounding Biases** | Considers the study's efforts to account for factors that could influence results (e.g. passive exposure), and selective reporting of information. | 0 | High risk: no consideration for potential confounders |
|  |  | 1 | Moderate risk: partial consideration of potential confounders with incomplete control methods or selective reporting |
|  |  | 2 | Low risk: comprehensive identification and control for all relevant confounding factors, no selective reporting |

*** 0**: High risk of bias; **1**: Some concerns; **2**: Low risk of bias.

**Thresholds for converting the Newcastle-Ottawa scales to Agency for Healthcare Research and Quality (AHRQ) standards (low risk, some concerns, high risk):**

- **0–2 Points**: High risk of bias - Indicates significant limitations in study design or methodology that raise concerns about the validity of the findings related to cocaine exposure.
- **3–4 Points**: Some concerns - Some methodological concerns or limitations are present, suggesting caution in interpreting the results.
- **5–6 Points**: Low risk of bias - Demonstrates a robust approach to studying cocaine exposure, with minimal risk of bias, using advanced detection methods and thorough control for confounding factors, enhancing confidence in the study's findings.
