# Supplementary material for: Children’s exposure to cocaine detected by hair analysis: a systematic review and meta-analysis
Source: BMC Pediatr. 2025 Oct 21;25:839. doi: 10.1186/s12887-025-06146-x (PMC12542512; doi:10.1186/s12887-025-06146-x)
Supplement: Supplementary file 3 — Additional File 3. Summary of included studies. [file 12887_2025_6146_MOESM3_ESM.docx]

**Table 1.** Summary of included articles.

| **First author, Year**  **Country** | **Age of children underwent hair analysis** | **Hypothesized or possible nature of the exposure** | **Length of analyzed hair** | **Children positive for cocaine in hair, N.** | **Cocaine concentration in hair (ng/mg):**  **Median (IQR)**  **[minimum-maximum]** | **Benzoylecgonine concentration in hair (ng/mg):**  **Median (IQR)**  **[minimum-maximum]** | **Additional information** |
| --- | --- | --- | --- | --- | --- | --- | --- |
| **Observational studies** | | | | | | | |
| DiGregorio, 1994  United States of America | Median (IQR): 0 (1) days  Range: 0-1 days | Intrauterine | Not specified | 22 | 16.80 (12.93)  [0.8-243.5] | 4.50 (5.15)  [0-55.4] | The study involved mothers with no prenatal care and their newborns. It found that women with a positive urinary drug screen by EMIT for benzoylecgonine had exposed their babies to cocaine. Among pregnant women with a negative drug screen for benzoylecgonine, 52.2% of their newborns had prior fetal exposure to cocaine. |
| Smith, 1996  United States of America | Median (IQR): 3 (4.5) years  Range: 1-13 years | Environmental exposure | Not specified | 23 | 0.94 (2.38)  [0.08-14.4] | 0.12 (0.84)  [0-5.4] | The study involved adult patients from drug rehabilitation programs, with a documented cocaine use disorder, and the children with whom they lived. In several households, children's hair showed higher levels of substance than adults’ hair. The findings were deemed consistent with environmental exposure and not ingestion in most children. |
| Vinner, 2003  France | Range: 0-2 days | Intrauterine | Not specified | 2 | < LOQ (2 ng/mg) and 17.9 | - | The study involved prenatal women, either under treatment with alternative medication or suspected to be addicted to illicit substances, and their newborns. New mothers (<24 h) without prenatal care but with admitted psychoactive drug addiction or suspected drug addiction were also included. The results of urine, meconium, and hair analysis were utilized to assess gestational drug exposure and to investigate the value in prediction of the appearance of neonatal withdrawal syndrome. |
| Joya, 2009  Spain | Mean (SD): 2.2 (1.3) years  Range: 18 months-5 years | Environmental exposure (e.g., parental use, accidental ingestion) | Minimum 4 cm - up to 6 cm | 21 | 1.57 (N.A.)  [0.3-5.96] | 0.85 (N.A.)  [0.2-1.4] | Hair samples were collected from children admitted to a pediatric emergency department. The study found that behavioral patterns with potentially harmful effects on the child's health (e.g., tobacco smoking, cannabis use, benzodiazepines and/or antidepressant consumption, and reduced breastfeeding duration) were significantly higher among parents of exposed children. A higher percentage of exposed children were in the lower weight percentile group compared with the nonexposed children. |
| Pragst, 2013  Germany | Range: 1-16 years | External contamination and passive inhalation or accidental ingestion | Up to 6 cm | 73 | <0.20 (<0.20)  [<0.20-17.80] | N.A. | The study involved children with parents under methadone substitution or suspected using illegal drugs. Within families, children’s hair and parents’ hair showed often the same drug pattern. Cocaine was the most frequently detected drug in children hair. Younger children were found having higher drug concentrations at hair analysis than elder siblings. |
|  | **Subset of children with cocaine in hair ≥ 0.20 ng/mg** | | | | | |  |
|  | Median (IQR): 2.0 (3.5)  Range: 1-7 years | External contamination and passive inhalation or accidental ingestion | Up to 6 cm | 19 | 1.22 (1.69) [0.2-17.80] | 0.093 (0.270)  [0.01-2.23] |  |
| Pichini, 2014 (1)  Spain | Range: 2-10 years | Passive or active exposure (e.g. parental use, contact, accidental ingestion) | 4 cm | 23 | 0.54 (0.48)  [0.15-3.81] | 0.18 (0.175)  [0.07-0.55] | This study was conducted in a pediatric emergency department in a low socioeconomic area. Children's exposure to drugs of abuse was significantly associated with higher maternal smoking habits, higher percentage of cannabis consumption by both parents, and higher maternal use of benzodiazepines and/or antidepressants. |
| Pichini, 2014 (2)  Spain | Median: 4 years | Passive exposure | 4 cm | 43 | 0.32 (N.A.)  [N.A.] | 0.26 (N.A.)  [N.A.] | The article describes three cohorts corresponding to three studies. One of these consists of children recruited in 1998 from the Asthma Multicenter Infant Cohort Study (AMICS), a study designed to investigate the effects of several pre- and post-natal environmental exposures on the inception of atopy and asthma. At 4 years of age, hair samples were collected and stored for eventual analysis of environmental exposure to several toxic substances, including cocaine. Hair samples of these children were then examined for the presence of drugs of abuse. The results of the three study cohorts suggested a significant prevalence of unsuspected pediatric exposure to drugs of abuse mainly involving cocaine. |
| Himes 2014  United States of America | 6.5 years | Environmental exposure | 3 cm | 3 *(No PME)* | 0.102 (N.A.)  [0.074-0.195] | 0.027 (N.A.)  [0.009-0.203] | The study enrolled mother-infant pairs where prenatal amphetamine exposure was determined by maternal self-report and/or positive meconium results. At the 6.5-year follow-up visit, children's hair was collected. Prenatal methamphetamine exposure, alone and in combination with postnatal drug exposures, was associated with behavioral and executive function deficits at 6.5 years. |
|  |  |  |  | 6 *(PME)* | 0.062 (N.A.)  [0.039-0.315] | 0.013 (N.A.)  [0.039-0.315] |  |
| Stauffer, 2015  United States of America | Median: 2.1 years  Range: 0-17.5 years | Environmental exposure and/or administration | Not specified | 37 | N.A. | N.A. | A retrospective chart review on cases of children underwent hair analysis in the context of child abuse evaluation was performed. Hair toxicology testing was positive for at least one compound in 17.2%. In 64% of cases hair testing identified the parent compounds without their respective tested metabolites, compatible with significant environmental exposure. |
| Alvarez, 2018  France | Range: 1 day-15 years | Not defined | Up to 8 cm | 2 | 0.016 and 0.763  (segments’ median) | 0 and 0.113, (segments’ median) | Children hospitalized for presumed intoxication were included. Hair was sampled within 1 to 15 days after intoxication or admission; the results of hair testing suggested that children aged less than approximately 29 months have hair so porous that the presence of drugs in sebum and sweat may lead to their incorporation into the hair shaft, resulting to the inefficacy of decontamination procedure. |
| Pragst, 2019  Germany | Range: 1-14 years | Passive exposure | Up to 6 cm | 61 | N.A. | N.A. | The study evaluated hair samples from 141 families with drug-consuming parents, focusing on the comparison between adults and their children. One to 5 drugs were detected in 239 (95.2%) of the family tests, being cocaine and THC the most frequently detected substances. The authors observed that the child/parent drug concentration ratio decreased with increasing children's age and was higher for boys than for girls. |
|  | **Subset of children > 7 years** | | | | | |  |
|  | Range: 7-10 years | Passive exposure | Up to 6 cm | 11 | N.A.  [0.03-0.56] | N.A. |  |
|  | Range: 11-14 years |  |  | 7 | N.A.  [0.02-1.24] | N.A. |  |
| Franz, 2020  Germany | Range: < 1 year | Intrauterine, breastfeeding, physical contact (babies). Passive and/or active exposure, accidental exposure | 1-6 cm | 6 | 0.07 (N.A.)  [0.01-5.40] | 0.01 (N.A.)  [0.01-0.71] | The study included hair samples of children whose caregivers had been identified as drug users at least in the past. The results of hair analysis suggested that with the increasing age of the child, the risk of being exposed to drug-consuming caregivers or environmental exposure decreases. The increase in drug concentration in adolescents’ hair suggests accidental or active use of the substances, possibly in addition to passive exposure. |
|  | Range: 1-<6 years |  |  | 31 | 0.15 (N.A.)  [0.02-20.0] | 0.02 (N.A.)  [0.01-2.90] |  |
|  | Range: 6-<14 years |  |  | 21 | 0.10 (N.A.)  [0.01-2.80] | 0.01 (N.A.)  [0.01-0.52] |  |
|  | Range: 14-16 years |  |  | 17 | 0.14 (N.A.)  [0.03-15.0] | 0.02 (N.A.)  [0.01-5.40] |  |
| Bertaso, 2023  Italy | Range: 0-1 days | Intrauterine | Not specified | 19 | 0.52 (1.31)  [0.05-38.6] | 0.43 (1.61)  [0-6.3] | The study evaluated cases of suspected in-utero drug exposure. The hair of mothers, neonates, and fathers (when available) was collected. When analyzing maternal and newborn hair, 92% tested positive, and > 50% of these were positive for more than one class of substance. Hair analysis was described as providing useful information about in utero exposure and on family background. |
| Garcia-Caballero, 2023  Spain | Range: 1 month-9 years | Accidental consumption, active administration | Up to 11 cm | 12 | 11.20 (13.43)  [0.25-28.67] | 0.63 (0.95)  [0.08-2.38] | Hair of children who were attended to in emergency services were analyzed. The results suggested that young children (1–3 years old) are more vulnerable to drug consumption environments. |
| Haas, 2023  Switzerland | Mean 14.9 years (SD +/- 0.85) | Active consumption | 3-5 cm | 5 [quantification reported only for 2 cases] | 0.044 and 1.5 | N.A. | In this prospective study, patients aged 16 years and younger admitted to the emergency department with alcohol intoxications were included. Almost half of the patients tested positive for other substances, mostly cannabis and stimulants. Patients’ statements and measured substances were consistent. |
| Cestonaro, 2024  Italy | Median (IQR): 6 (52) days  Range: 0-1 year | Intrauterine, breastfeeding, passive inhalation, accidental ingestion, intentional administration | Up to 6 cm | 42 | 0.655 (5.743)  [0.013-290] | 0.13 (2.45)  [0.001-23.8] | Hair samples of children aged 0-1 years and of their mothers were analyzed. The study revealed a benzoylecgonine/cocaine ratio reducing as children grow older. A child-to-mother cocaine concentration ratio lower than 1 was found in pairs where newborn was within a week old, whereas a ratio equal to or greater than 1 was found in pairs where infants were older. |
| Cestonaro, 2025  Italy | Range: 0-16 years | Intrauterine, breastfeeding, passive inhalation, accidental ingestion, intentional administration, active use | Median 3 cm (1-11) | 94 | 0.175 (1.039) [0.004-290] | 0.040 (0.263) [0.001-23.8] | Reports of hair analysis of children aged 1-16 years were analyzed. The highest rates of positives were found in young children within 3 years of age. The results suggest that exposure to drugs of abuse represents a non-negligible problem particularly in infants and toddlers. |
|  |  |  |  | **Subset of children > 1 year** | | |  |
|  |  |  |  | 52 | 0.090 (0.390)  [0.004-15.00] | 0.025 (0.054)  [0.001-1.08] |  |
| **Case reports** | | | | | | | |
| De Giorgio, 2004  Italy | 6 years | Environmental exposure | 1.5 cm | 1 | 16.0 | 0.6 | The child was admitted to the emergency department for general distress. Urine and hair samples showed were positive for cocaine. According to the authors, the findings most likely indicated that the child had passively consumed the drug when living in a heavily contaminated environment. |
| Garcia-Algar, 2005  Spain | 11 months | Passive exposure or ingestion or intentional administration or breast milk | 8 cm | 1 | 2.95 | 0.45 | A 11-month-old infant was admitted to the emergency department with apparent generalized seizures. Urine tested positive for MDMA. Segmental hair analysis also revealed chronic exposure to cocaine. Drug consumption by the mother could not be objectively investigated as she submitted only bleached scalp hair. |
| Papaseit, 2010  Spain | 13 months | Environmental exposure, passive inhalation, active ingestion | 1 cm | 1 | 17.24 | 0.6 | An infant of parents undergoing methadone treatment presented with symptoms consistent with acute intoxication by methadone. Hair analysis revealed also exposure to cocaine. |
| Joya, 2011  Spain | 1 month | Intrauterine, breastfeeding, skin contact, passive inhalation | 2 cm | 1 | 17.5 | 2.2 | An infant was admitted to the emergency department with respiratory distress. The baby's mother admitted to consuming cannabis and beer the night before breastfeeding and later mentioned the possible ingestion of acetaminophen-codeine tablets by the baby. Opiates and cocaine were detected in baby’s hair. |
| Franz, 2020 | 1 year | External contamination | 1- 6 cm | 1 | 3.25 (0.052 six months after) | 0.105 (0.027 six months after) | Hair of five siblings were found positive for cocaine, amphetamines, and THC. Six months later, only one child tested positive for cocaine, while concentrations of THC increased in all siblings’ hair. These results could be explained by the change in the children's caregiving environment. A decreasing concentration with increasing age was observed for nearly all drugs under investigation, probably resulting from closer physical contact and less resistant cuticle of the hair at a young age. |
|  | 1 year |  |  | 1 | 0.607 (negative six months after) | 0.03 (negative six months after) |  |
|  | 5 years |  |  | 1 | 1.28 (negative six months after) | 0.063 (negative six months after) |  |
|  | 6 years |  |  | 1 | 0.154 (negative six months after) | 0.013 (negative six months after) |  |
|  | 8 years |  |  | 1 | 0.088 (negative six months after) | 0.007 (negative six months after) |  |

**Abbreviations**: PME = Prenatal methamphetamine exposure; IQR = Interquartile range; LOQ = Limit of quantification; MDMA = 3,4-Methyl​enedioxy​methamphetamine; SD = Standard deviation; THC = Tetrahydrocannabinol.
