# Supplementary figures and images for: Children’s exposure to cocaine detected by hair analysis: a systematic review and meta-analysis
Source: BMC Pediatr. 2025 Oct 21;25:839. doi: 10.1186/s12887-025-06146-x (PMC12542512; doi:10.1186/s12887-025-06146-x)

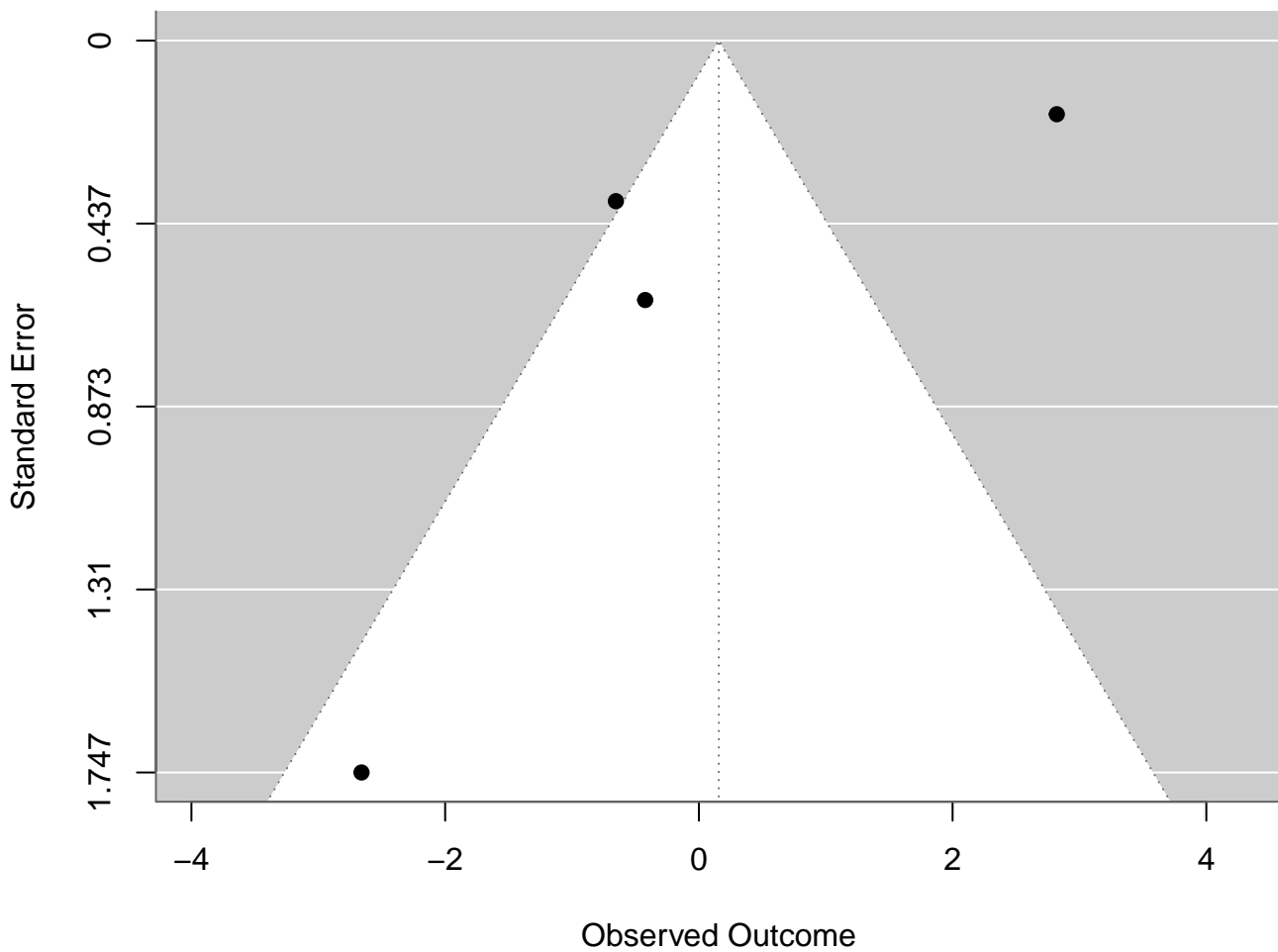

Supplement: Supplementary file 4 — Additional File 4. Publication bias assessment – Funnel Plot, Group A. [file 12887_2025_6146_MOESM4_ESM.pdf]

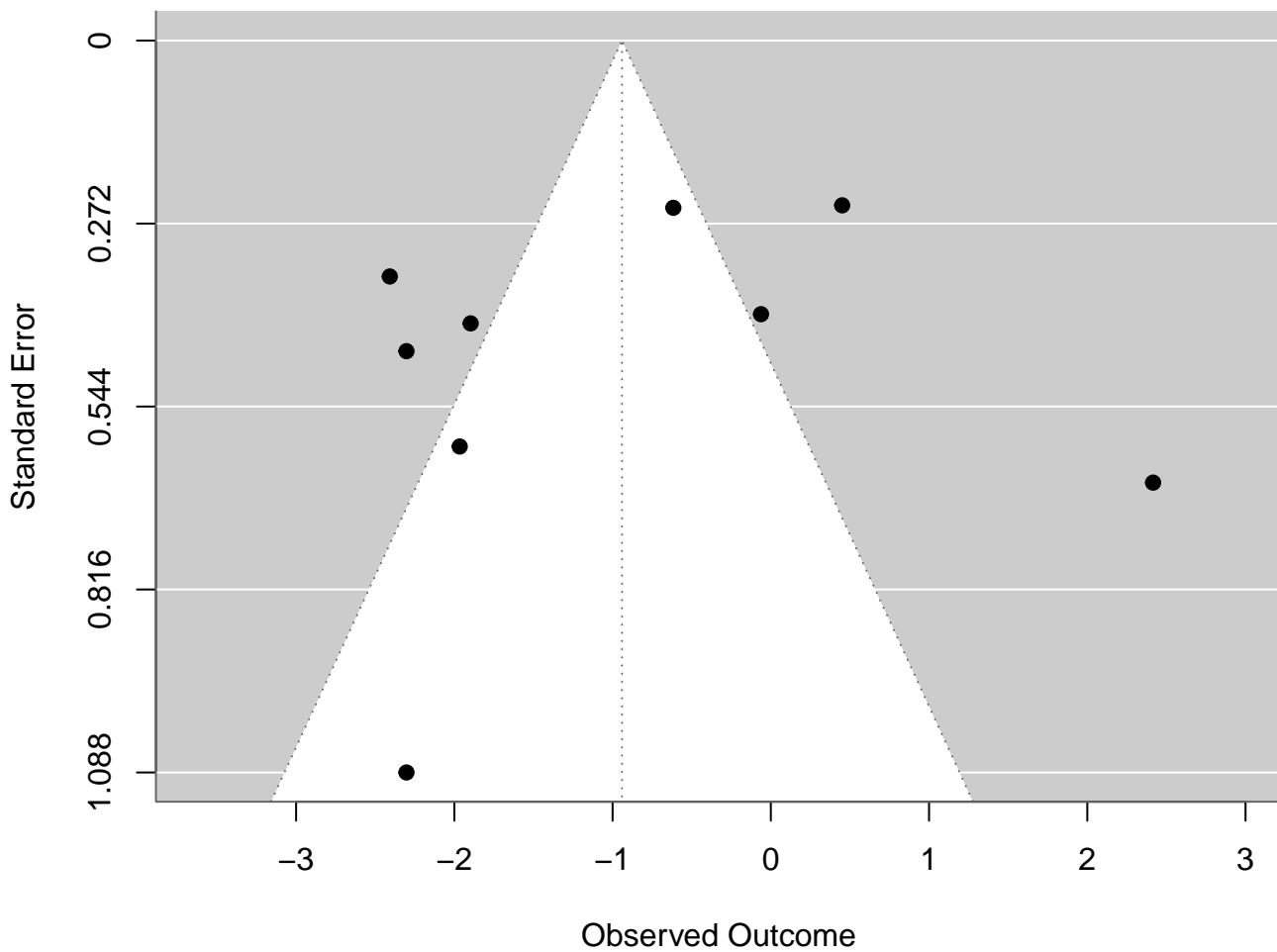

Supplement: Supplementary file 5 — Additional File 5. Publication bias assessment – Funnel Plot, Group B. [file 12887_2025_6146_MOESM5_ESM.pdf]

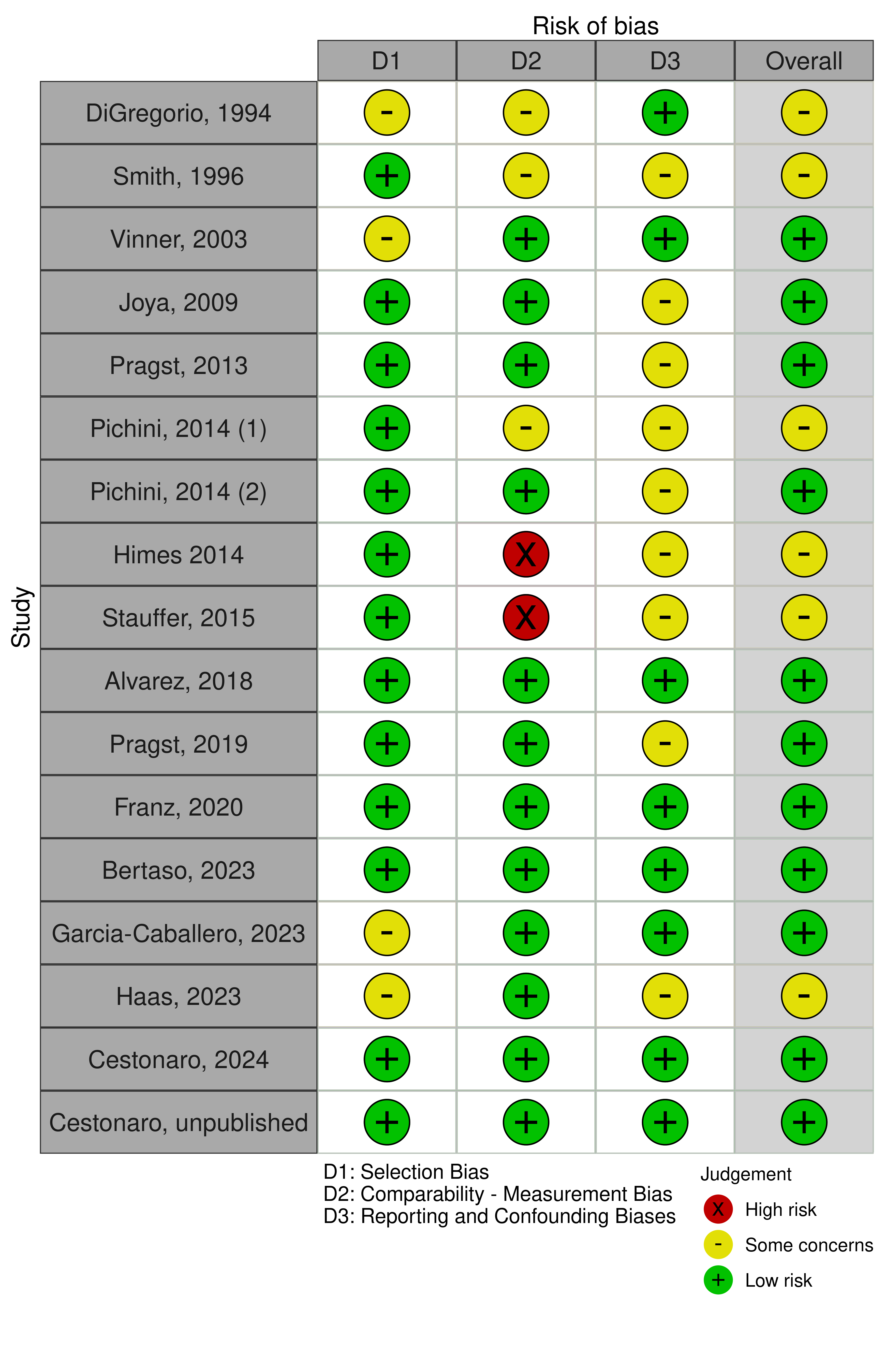

Supplement: Supplementary file 6 — Additional File 6. Risk of bias assessment – Traffic Light Plot (.png). [file 12887_2025_6146_MOESM6_ESM.png]

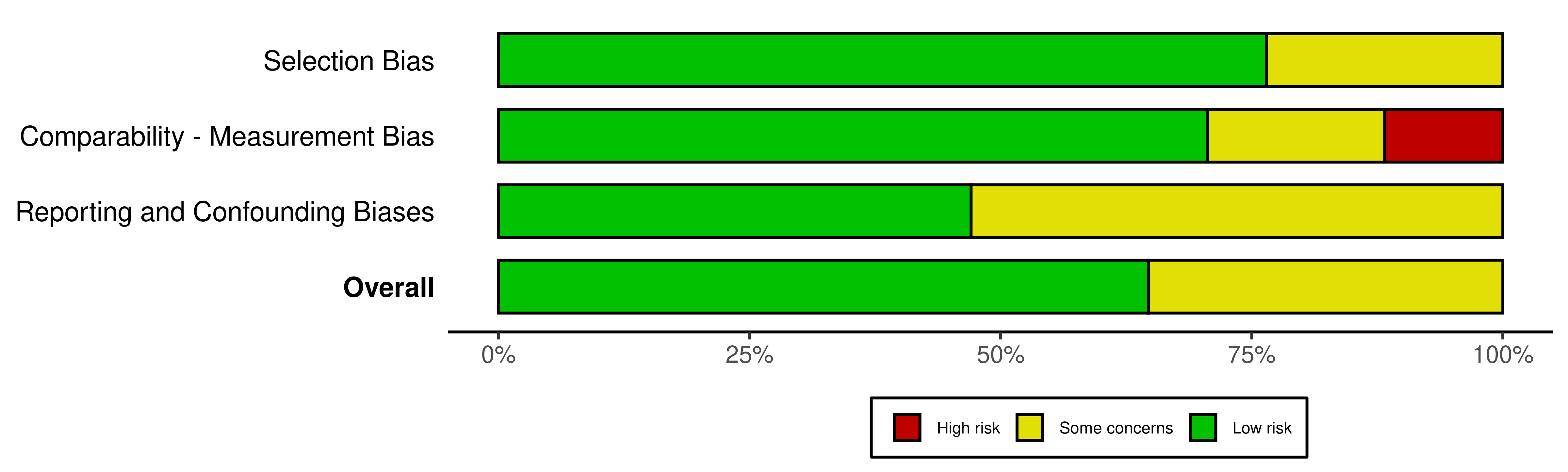

Supplement: Supplementary file 7 — Additional File 7. Risk of bias assessment – Summary Plot (.png). [file 12887_2025_6146_MOESM7_ESM.png]

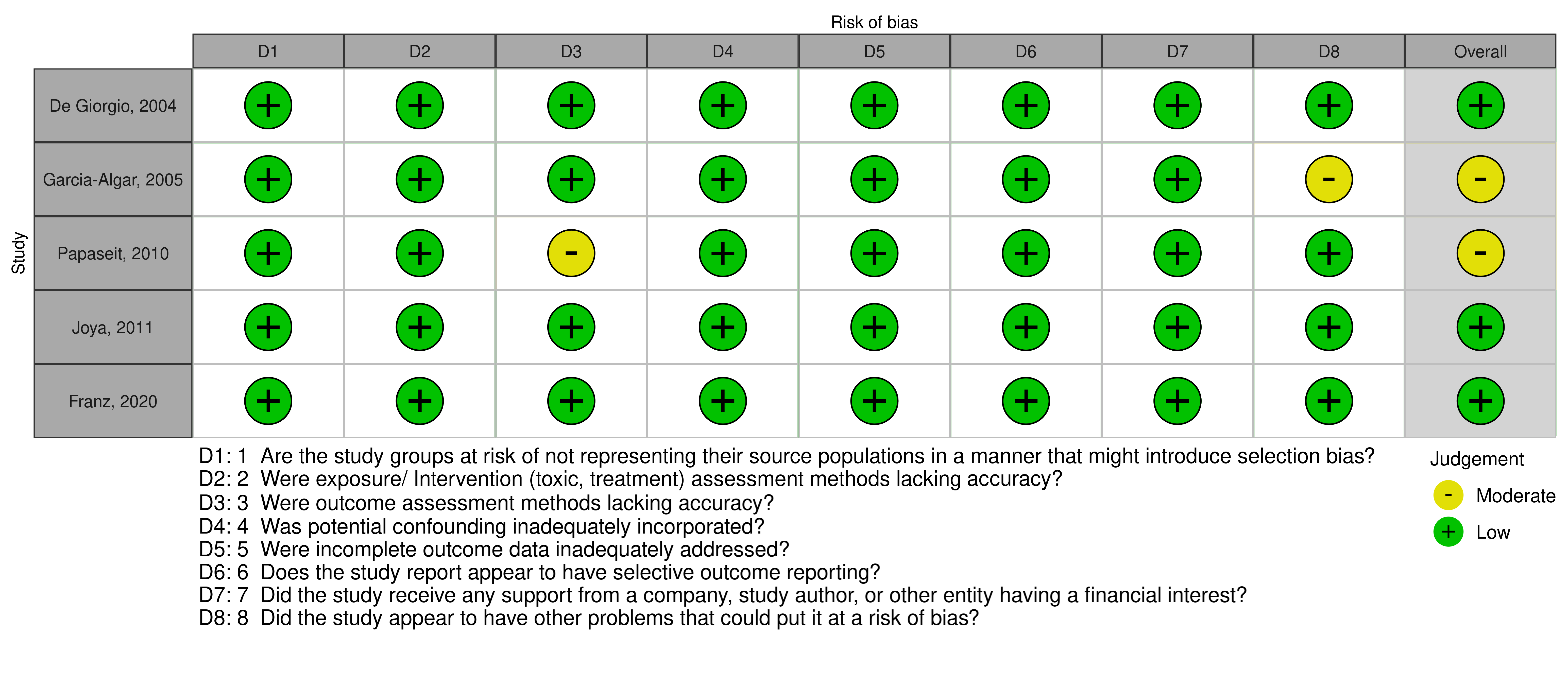

Supplement: Supplementary file 8 — Additional File 8. Risk of bias assessment – Traffic Light Plot (.png). [file 12887_2025_6146_MOESM8_ESM.png]

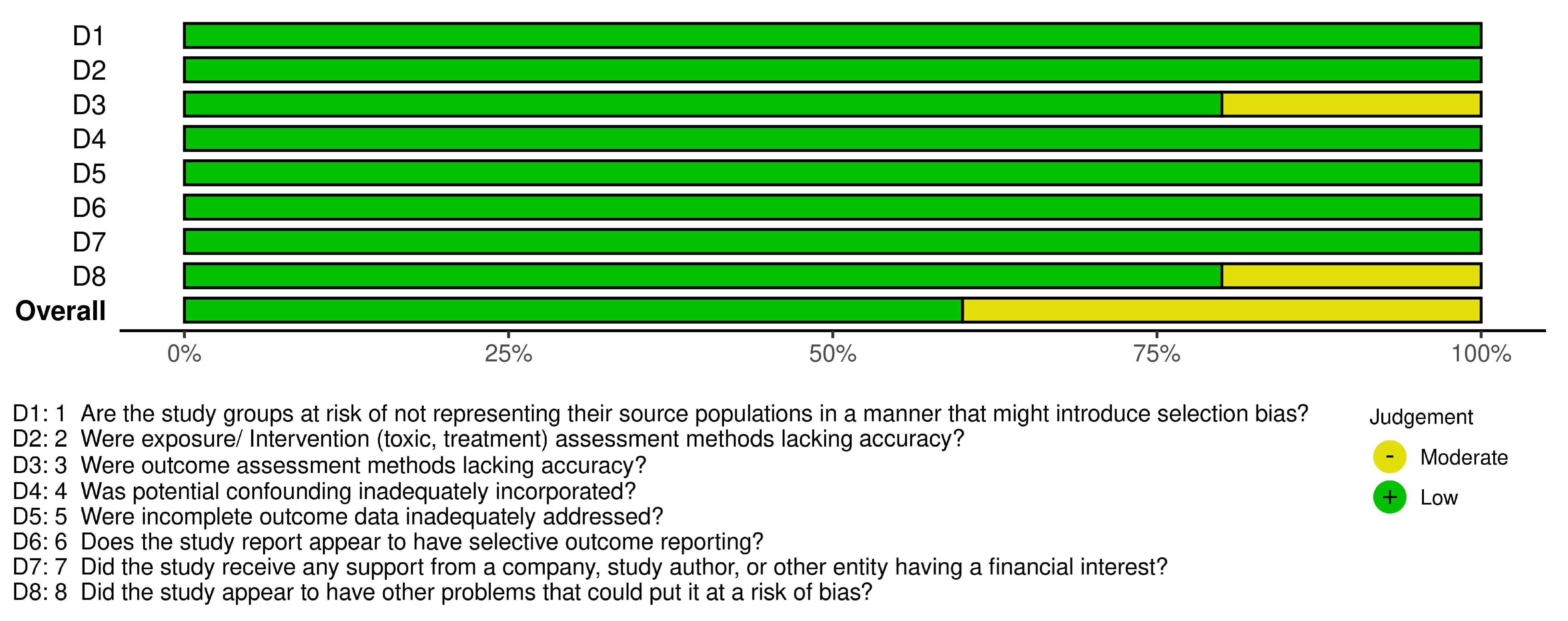

Supplement: Supplementary file 9 — Additional File 9. Risk of bias assessment – Summary Plot (.png). [file 12887_2025_6146_MOESM9_ESM.png]
